# Influence of oil, dispersant, and pressure on microbial communities from the Gulf of Mexico

**DOI:** 10.1038/s41598-020-63190-6

**Published:** 2020-04-27

**Authors:** Nuttapol Noirungsee, Steffen Hackbusch, Juan Viamonte, Paul Bubenheim, Andreas Liese, Rudolf Müller

**Affiliations:** Hamburg University of Technology, Institute of Technical Biocatalysis, Hamburg, 21073 Germany

**Keywords:** Microbial ecology, Water microbiology

## Abstract

The *Deepwater Horizon* incident in the Gulf of Mexico in 2010 released an unprecedented amount of petroleum hydrocarbons 1500 meters below the sea surface. Few studies have considered the influence of hydrostatic pressure on bacterial community development and activity during such spills. The goal of this study was to investigate the response of indigenous sediment microbial communities to the combination of increased pressure, hydrocarbons and dispersant. Deep-sea sediment samples collected from the northern Gulf of Mexico were incubated at atmospheric pressure (0.1 MPa) and at elevated pressure (10 MPa), with and without the addition of crude oil and dispersant. After incubations at 4 °C for 7 days, *Colwellia* and *Psychrobium* were highly abundant in all samples. Pressure differentially impacted members of the Alteromonadales. The influences of pressure on the composition of bacterial communities were most pronounced when dispersant was added to the incubations. *Moritella* and *Thalassotalea* were greatly stimulated by the addition of dispersant, suggesting their roles in dispersant biodegradation. However, *Moritella* was negatively impacted by increasing pressure. The presence of dispersant was shown to decrease the relative abundance of a known hydrocarbon degrader, *Cycloclasticus*, while increasing pressure increased its relative abundance. This study highlights the significant influence of pressure on the development of microbial communities in the presence of oil and dispersant during oil spills and related response strategies in the deep sea.

## Introduction

The explosion of the *Deepwater Horizon* (DWH) oil drilling platform in 2010 led to the release of 700,000 metric tons of crude oil into the Gulf of Mexico at the water depth of 1500 m^1,2^. The subsurface release of oil formed a persistent plume spanning 1000 and 1200 m depth^3,4^ and analysis of deep sediment cores collected near the blowout location shortly after the spill indicated that some of this oil was ultimately deposited on the sea floor^5,6^. The deposition of these hydrocarbons were from marine oil snow sedimentation and flocculent accumulation (MOSSFA) events, where crude oil compounds were attached to sinking of particles^7^, and from the direct contact of the deep plume with the continental shelf, referred to as the bathtub-ring hypothesis^6^. Unlike the archaeal community, the bacterial community exhibited a measured response to the massive input of hydrocarbons from the DWH event^8^. Marine oil-degrading bacteria responded with increased abundances in the presence of crude oil. Half-lives of dispersed oil in aerobic marine waters varied from days to months and were influenced by various factors including pre-adaptation of microbial communities to hydrocarbons and the availability of nutrients essential for microbial growth and biodegradation (nitrogen, phosphorous, or iron)^9^. It is estimated that the portion of oil degraded by the bacterial community was as high as 61%^5^. A number of studies investigated the successions of the bacterial community compositions in the water column and in the sediments^10–13^ and found that these successions were driven by the hydrocarbons input from the DWH spill^10,12,14^.

One of the response strategies employed during the spill was the subsea application of dispersant, Corexit EC9500A, which was directly injected at the wellhead during the spill^1^. The effectiveness of dispersant on marine oil biodegradation is a subject of debate^15,16^. One study suggested that dispersant inhibited hydrocarbon-degrading *Marinobacter*, but stimulated dispersant-degrading *Colwellia*^16^. A similar study suggested that dispersant enhanced oil biodegradation^15^.

The deep sea is a unique environment, where the hydrostatic pressure increases linearly with depth (1 MPa per 100 m). Previous studies on the influence of pressure on oil biodegradation^17–20^, showed that pressure as low as 5 MPa impaired growth and activity of hydrocarbon-degrading *Alcanivorax borkumensis*^18^ and the growth of naphthalene degrading *Sphingobium yanoikuyae* was inhibited at 8.8 MPa^21^. A recent study demonstrated a 4% decrease in n-alkane biodegradation for every 1 MPa increase in hydrostatic pressure^22^. In general, investigations on the impact of dispersant on hydrocarbon biodegradation were performed at ambient pressures, overlooking the effect of pressure^15,23–27^. More recently, growth of *Rhodococcus* isolated from deep-sea sediment has been shown to be impaired in the presence of dispersant at 15 MPa^28^. However, biodegradation studies with crude oil and dispersants at increased pressures have not yet been conducted on benthic bacterial communities. In order to understand how each of these factors influenced deep-sea bacterial communities, investigations under controlled *ex-situ* conditions were conducted. Here we report how the interactions between crude oil, pressure, and dispersant application changed deep benthic microbial communities around the DWH region.

## Results

### Analysis of microbial diversity

#### Alpha diversity

**Figure 1:**
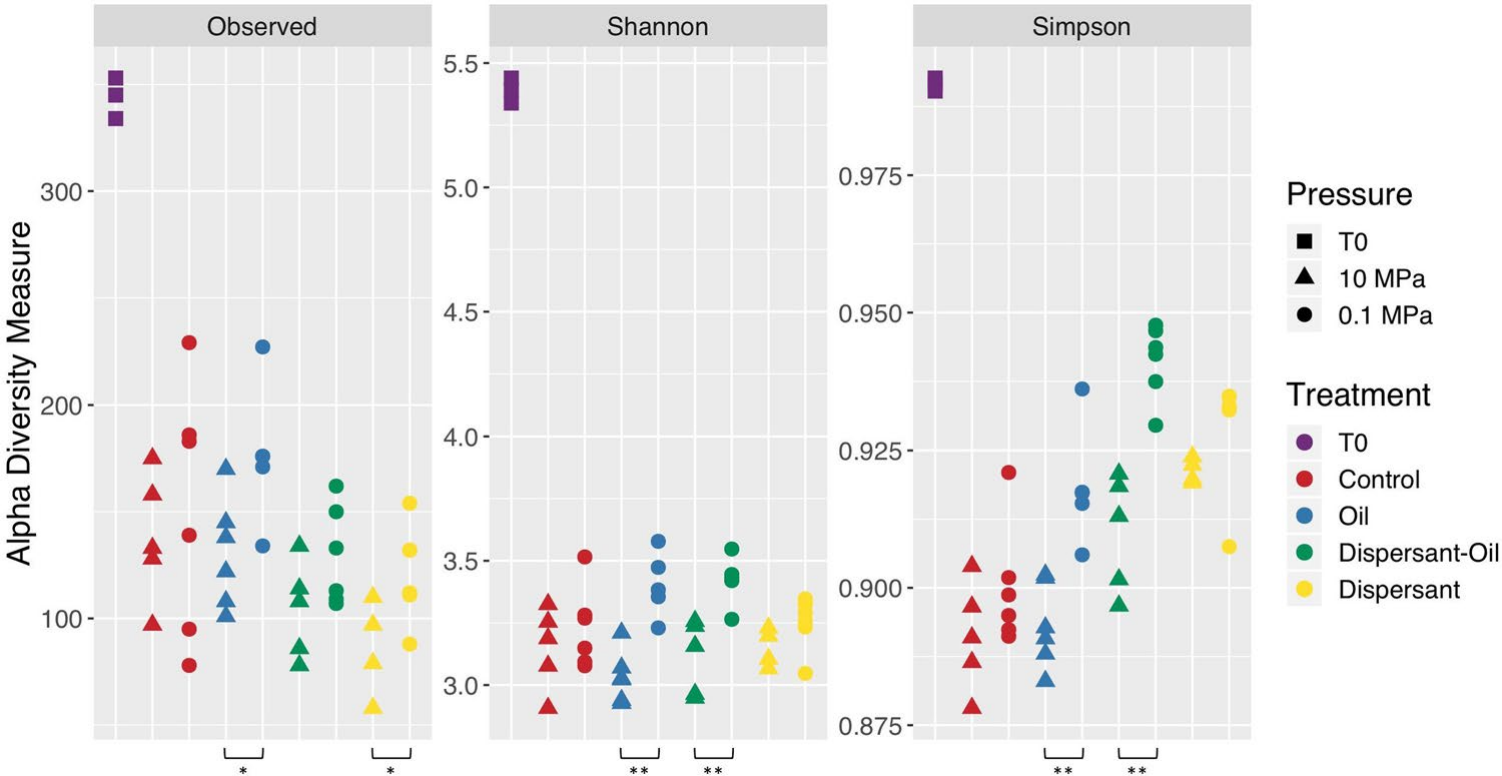
Alpha diversity. Observed species, Shannon and Simpson diversity results are grouped by treatment. T0 (initial community) are colored in purple, Control group (no oil or dispersant added) are colored in red, Incubations with oil are colored in blue, Incubations with dispersant and oil are colored in green, and incubations with dispersant are colored in yellow.Incubations at 10 MPa are represented by triangles, at 0.1 MPa are represented by circles. Wilcoxon rank sum test: *(p < 0.05) **(p < 0.01).

The alpha diversity was determined for each treatment and pressure scenario before and after incubation. After 7 days of incubation for all treatments and pressures, there was a decline in alpha diversity (Fig. 1). The control, without addition of oil or dispersant at ambient pressure, lost more than half of the diversity after incubation. Pressure did not affect the alpha diversity of control groups (no oil or dispersant added). The number of observed species, the Shannon diversity index and the Simpson diversity index of the control group were not significantly different at 0.1 MPa and 10 MPa (Fig. 1). The addition of oil and/or dispersant led to decreases in alpha diversities at both 0.1 MPa and 10 MPa. Observed numbers of species in the incubation with dispersant were lower at 10 MPa than at 0.1 MPa. Alpha diversities of the sediment communities treated with oil at 0.1 MPa were significantly different from those at 10 MPa. Observed numbers of species, Simpson indices, and Shannon indices of 10 MPa incubations were lower than those of 0.1 MPa incubations (Fig. 1). Although the observed species of the communities incubated with oil and dispersant at 0.1 MPa and 10 MPa were not different, the presence of oil consistently resulted in statistically significant decreases in alpha diversity when the communities incubated at 0.1 MPa and 10 MPa were compared.

#### Beta diversity

**Figure 2:**
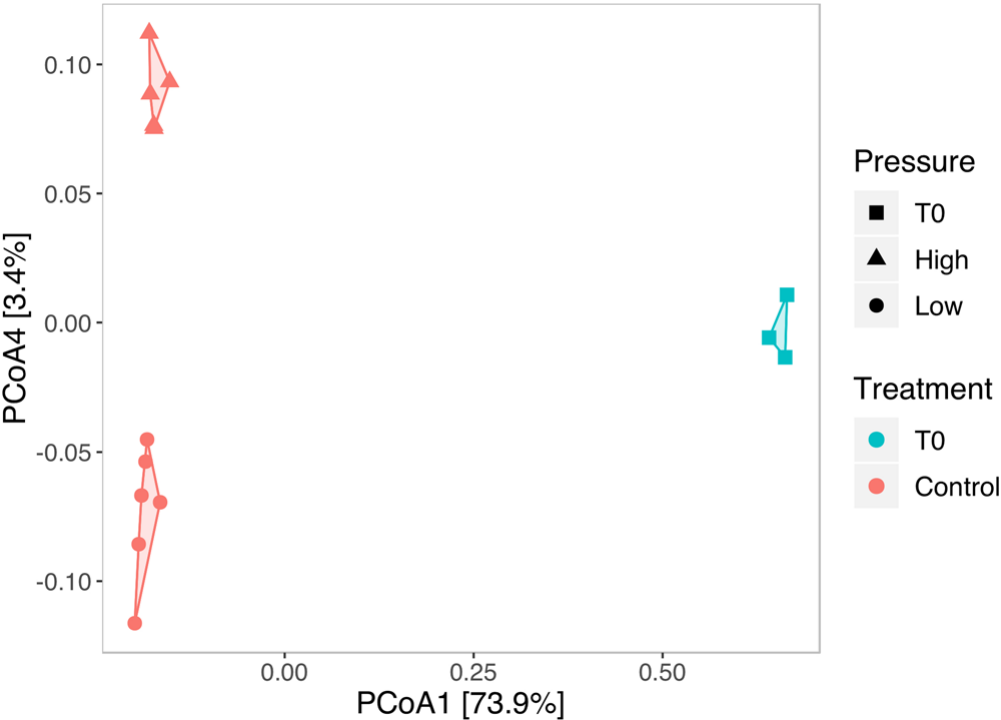
Principal Coordinate Analysis (PCoA) of Bray-Curtis dissimilarities between the initial communities before incubation and controls without addition of oil or dispersant after incubation for 7 days. Initial community pressures are represented by squares. Incubations at 10 MPa are represented by triangles, at 0.1 MPa are represented by circles.

**Figure 3:**
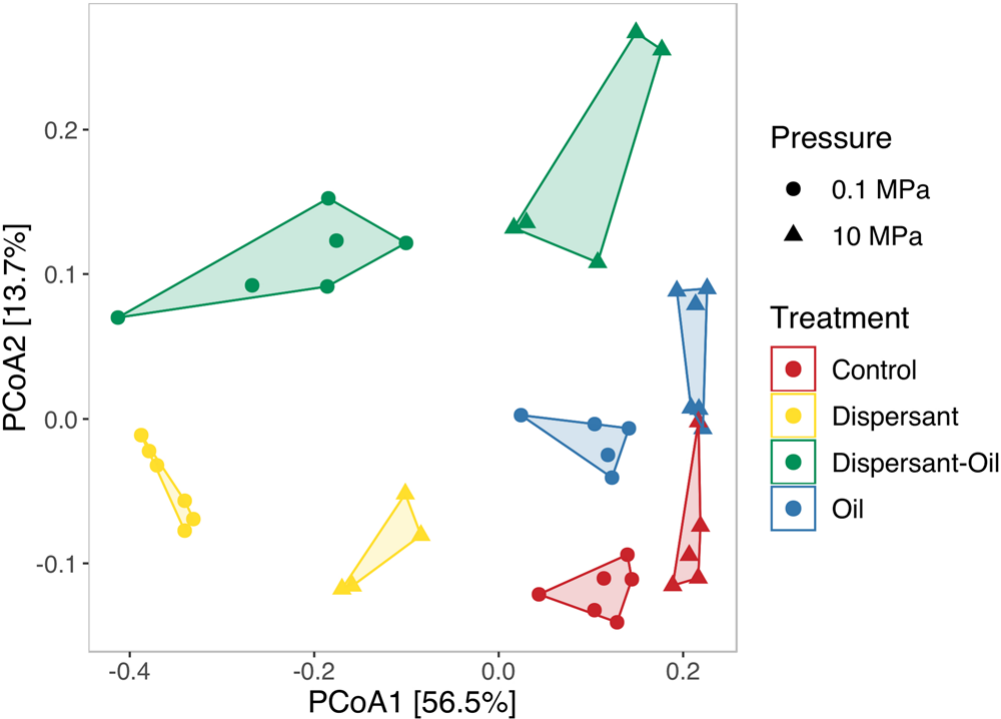
Comparison of microbial community structures. Principal Coordinate Analysis (PCoA) of 16S rRNA gene sequences using Bray-Curtis distance grouped by treatment. Control group (no oil or dispersant added) are colored in red, Incubations with oil are colored in blue, Incubations with dispersant and oil are colored in green, and incubations with dispersant are colored in yellow. Incubations at 10 MPa are represented by triangles, at 0.1 MPa are represented by circles.

The incubation of sediment slurries without the addition of oil or dispersant for 7 days caused significant shifts of microbial compositions at 0.1 MPa (p = 0.017, R^2^ = 0.77, PERMANOVA, N = 9) and at 10 MPa (p = 0.012, R^2^ = 0.7513, PERMANOVA, N = 8) compared to the initial communities, reflecting a shift of microbial communities influenced by incubation conditions. The microbial communities of the control groups incubated at 0.1 MPa were different from those incubated at 10 MPa (p = 0.002, R^2^ = 0,285., PERMANOVA, N = 11) (Fig. 2). The sediment community composition was changed by the presence of oil (p = 0.001, R^2^ = 0.118, PERMANOVA, N = 43), dispersant (p = 0.001, R^2^ = 0.370, PERMANOVA, N = 43) and pressure (p = 0.001, R^2^ = 0.131, PERMANOVA, N = 43). Hydrostatic pressure exerted a higher influence on microbial communities when oil and/or dispersant were added into the incubation (Table 1). Principal Coordinate Analysis (PCoA) using a Bray-Curtis distance showed the effect of treatment and pressure on microbial community dynamics (Fig. 3).

**Table 1:** Pair-wise PERMANOVA of Bray-Curtis distances.

Comparison (0.1 MPa and 10 MPa)		
Treatment	R^2^	p-value
Control	0.28552	0.002
Dispersant	0.66594	0.002
Dispersant-Oil	0.48354	0.006
Oil	0.38667	0.004

Communities after incubation of sediment in different treatments at 0.1 MPa were compared to those at 10 MPa. Statistical analysis was performed on Bray-Curtis distances with permutational multivariate analysis of variances (PERMANOVA) with 999 permutations.

#### Microbial community structure changes in response to pressure

**Figure 4:**
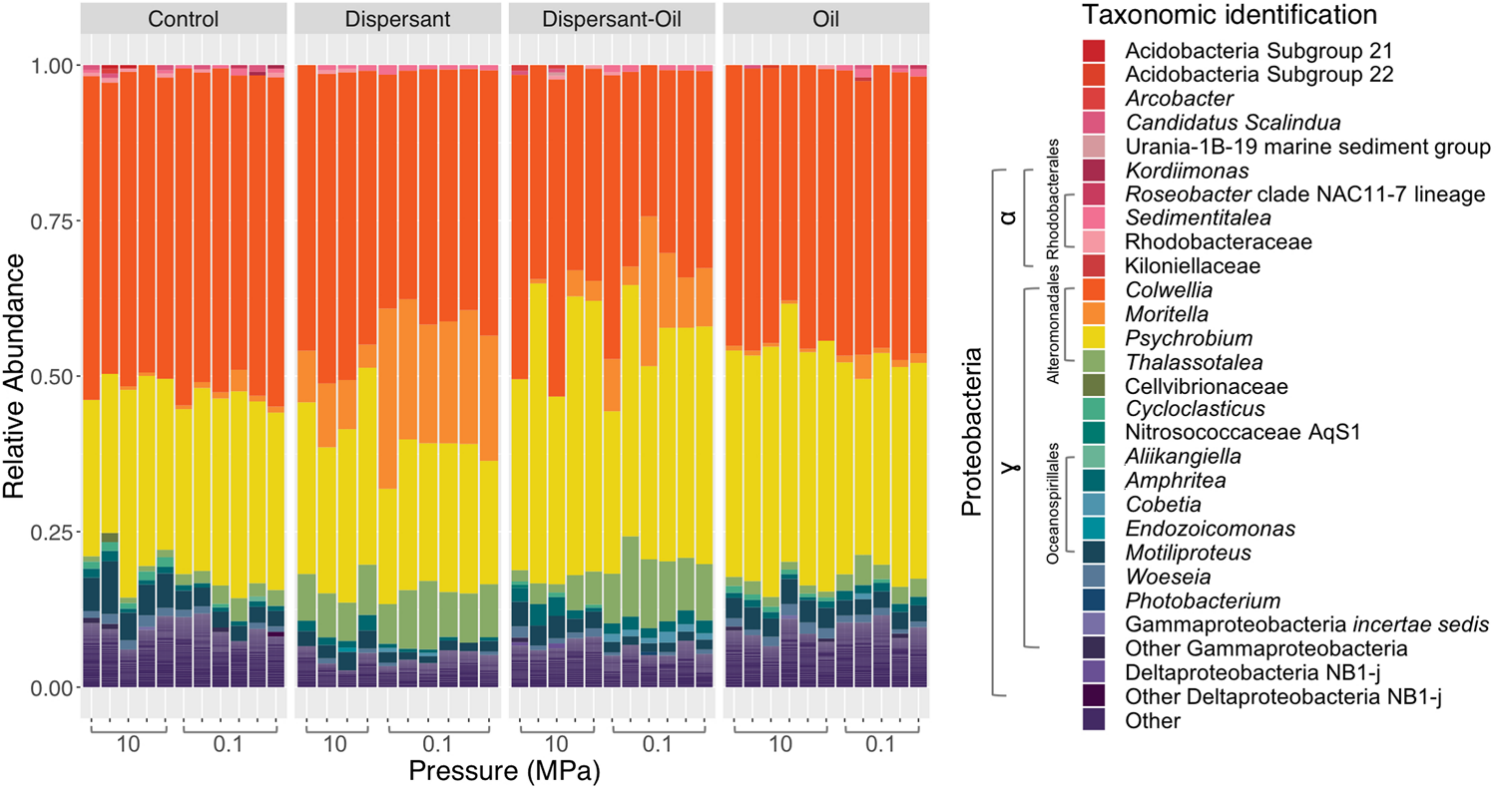
Relative abundance plots showing the response of the microbial community to different treatments and pressures. Treatments were Control (no dispersant or oil addition), Dispersant (dispersant only added), Dispersant-Oil (dispersant and oil added), and Oil (oil added). Taxa shown were grouped at genus level. Each single bar represents one biological replicate.

The phylum Proteobacteria increased in relative abundance in controls and all treatments after the incubation. Planctomycetes was present in all treatments with a maximum contribution of 2.5% of the total number. Bacteroidetes, Acidobacteria and Chloroflexi contributed to less than 0.1% of the population in all treatments after incubation. Gammaproteobacteria was the dominant class of Proteobacteria comprising more than 90% of the community in controls and in all treatments after incubation (Supplementary Fig. S2). Among the Gammaproteobacteria, Alteromonadales and Oceanospirillales were the most abundant bacterial orders. Colwelliaceae and Shewanellaceae were the two major families detected in all incubations (Fig. 4).

**Figure 5:**
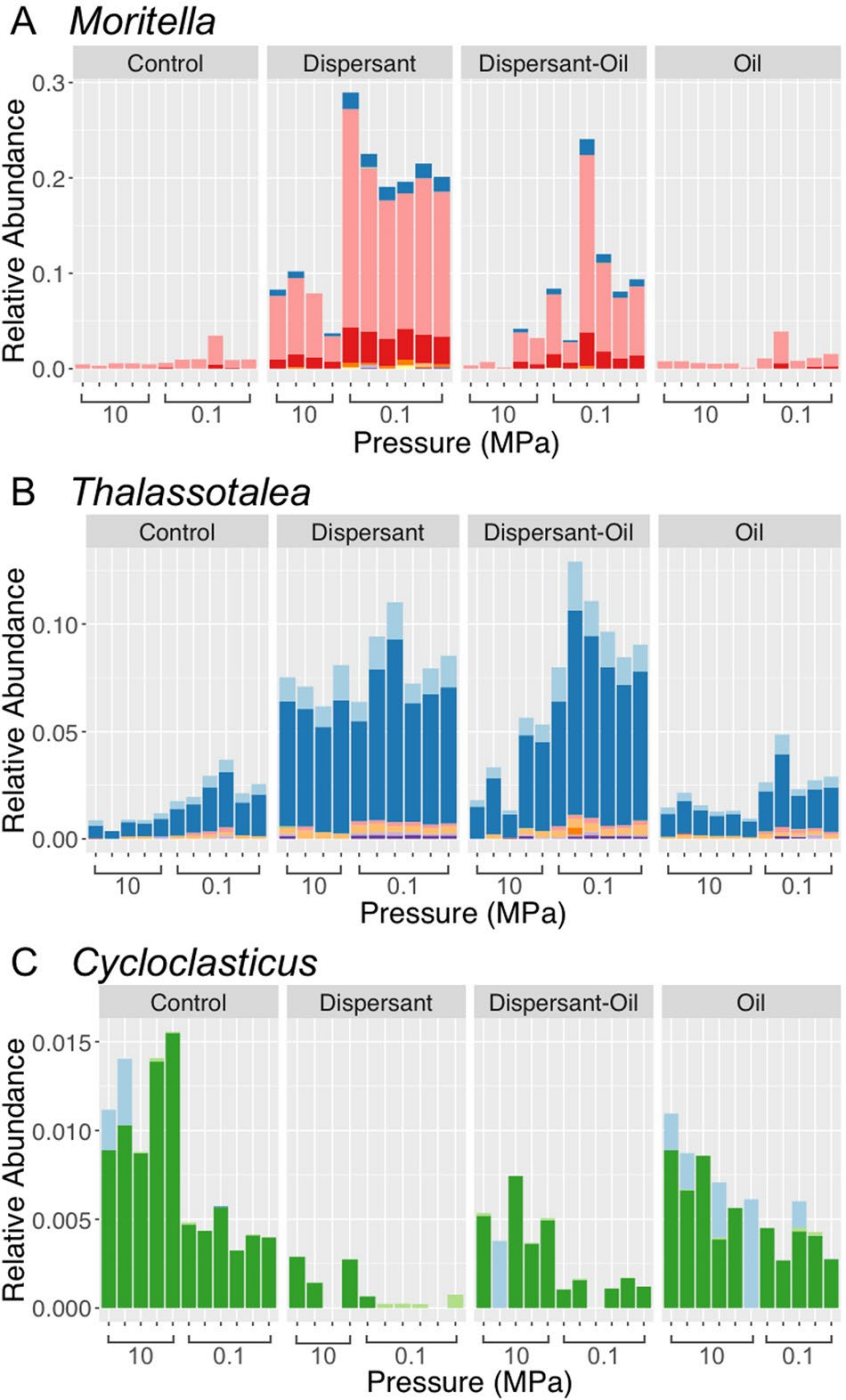
Relative abundance plots showing the response of microbial genera to different treatments and pressures. (**A**) *Moritella* (**B**) *Thalassotalea* (**C**) *Cycloclasticus*. Treatments were Control (no dispersant or oil addition), Dispersant (dispersant only added), Dispersant-Oil (dispersant and oil added), and Oil (oil added). Taxa shown were grouped at genus level. Each single bar represents one biological replicate. Different colors in each genus represent different ASVs belonging to the genus.

Pressure differentially affected bacterial taxa across taxonomic groups. Sequences related to the genus *Moritella* (Moritellaceae) responded negatively to pressure in the presence of dispersant. In the control and oil-treated groups, *Moritella*’s relative abundances reached up to 3.5% and 3.9% at 0.1 MPa, and 0.5% and 0.7% at 10 MPa, respectively. In dispersant-treated incubations, sequences related to the genus *Moritella* were more abundant at 0.1 MPa (19.0–28.9%) than at 10 MPa (3.7–10.2%). In the presence of both oil and dispersant, the genus’s relative abundance was also higher at 0.1 MPa (3.0–24.0%) than at 10 MPa (0.1–4.2%) (Fig. 5A).

*Thalassotalea* did not exhibit a significant response to pressure application with a dispersant-only treatment. The relative abundances were 6.4–11.0% at 0.1 MPa, and 6.2–8.1% at 10 MPa. However, *Thalassotalea* did respond to an increase in pressure when both oil and dispersant were present, in which the relative abundances were higher at 0.1 MPa (8.0–13.0%) than at 10 MPa (1.3–5.6%) (Fig. 5B), showing a decrease with increasing pressure.

Sequences belonging to the genus *Cycloclasticus* (Cycloclasticaceae) showed an increase in relative abundance with an increase in pressure to 10 MPa in all treatments. In the control groups, the maximum relative abundance at 10 MPa was three times of that at 0.1 MPa. In the oil-only treatment, the maximum relative abundance at 10 MPa was approximately double that at 0.1 MPa (1.1% at 10 MPa and 0.6% at 0.1 MPa). However, in the dispersant-only treatment, the maximum relative abundances of *Cycloclasticus* were 0.3% at 10 MPa and 0.07% at 0.1 MPa. However, in the presence of both oil and dispersant, the maximum relative abundance were 0.7% at 10 MPa and 0.17% at 0.1 MPa (Fig. 5C).

#### Differential abundance analysis

The control incubations at both 0.1 MPa and 10 MPa were dominated by the genera *Colwellia* (Colwelliaceae) and *Psychrobium* (Shewanellaceae). In order to identify the amplicon sequence variants (ASVs) that responded solely to pressure, differential abundance analysis was performed. This analysis resulted in 7 ASVs were differentially abundant at 10 MPa; 3 of which belonged to the genus *Motiliproteus* (Nitrincolaceae, Oceanospirillales). The other 4 ASVs were classified as uncultured Rhodobacteraceae (Rhodobacterales), *Psychrobium* (Shewanellaceae), *Colwellia* (Colwelliaceae), and *Cycloclasticus* (Cycloclasticaceae). Comparisons between the control incubations at 10 MPa and the oil incubations at 10 MPa were conducted in order to identify changes that were due to hydrocarbon addition at high pressure. Five ASVs, which were differentially abundant in incubation with oil at 10 MPa, belonged to the genus *Psychrobium*. There were 2 *Colwellia* ASVs and 1 *Thalassotalea* ASV that increased in abundance due to oil addition at 10 MPa.

The ASVs that were higher in abundance in the treated groups were classified as treatment responders. Subsequently, all of the treatment responders were subjected to Venn diagram analysis (Supplementary Fig. S1). In order to identify the ASVs that responded to oil and dispersant at 0.1 and 10 MPa, the microbial communities of treatment groups were first compared to control groups at 0.1 MPa or 10 MPa to identify those ASVs that did not respond specifically to the treatment. Those ASVs that were upregulated by oil were classified as oil responders. The oil responders dominantly consisted of the genus *Psychrobium* at both 0.1 MPa and 10 MPa pressure scenarios. Three ASVs of *Psychrobium* were enriched by oil at 10 MPa. The dispersant responders were more diverse, including members of Alteromonadales (*Moritella*, *Colwellia*, *Thalassotalea*, *Psychrobium*, and *Alkalimarinus*), Oceanospirillales (*Endozoicomonas*, *Cobetia*, and *Motiliproteus*), and Rhodobacterales (*Roseobacter*). A number of ASVs belonging to the genera *Psychrobium*, *Colwellia*, *Moritella*, *Alkalimarinus*, *Cobetia*, and *Motiliproteus* were found to be enriched by dispersant at 0.1 MPa, while the genera *Thalassotalea*, *Endozoicomonas*, and *Roseobacter* were enriched by dispersant at higher pressures of 10 MPa. The ASVs that were exclusively enriched in the oil plus dispersant treatment were categorized as “dispersed oil” responders. They contained mostly sequences related to the order Oceanospirillales including *Cobetia*, *Amphritea* and *Motiliproteus*. However, there was no sequence that was specifically enriched by dispersed oil at 10 MPa (Supplementary Table S1).

## Discussion

In this study, microbial communities incubated with dispersant and/or crude oil at two different pressures, 0.1 MPa and 10 MPa, exhibited different responses, suggesting that pressure influenced the succession of microbial communities. The effect of pressure was more conspicuous in the presence of external substrates (dispersant and crude oil) suggesting that hydrostatic pressure is an important parameter that influences how microbial communities respond to an oil spill.

The increase in the relative abundance of *Colwellia* and *Psychrobium* in all treatments, including the control, (Figs. 1 and 3) suggested that these genera favored the incubation conditions and were able to thrive on the dissolved organic material that was present in the sample without an additional hydrocarbon source. The members of Alteromonadales, including *Colwellia* and *Psychrobium*, have previously been shown to be enriched in unamended long-term incubations at high pressure and low temperature^29^, and an addition of dissolved organic material could increase growth of *Colwellia* and *Psychrobium*^30^.

We used differential abundance analysis to detect variants that responded specifically to crude oil or dispersant. The results showed that 6 ASVs belonging to the genus *Colwellia* were dispersant responders at 0.1 MPa. The role of *Colwellia* in the pelagic community has been studied extensively, and it has been suggested that *Colwellia* increased in abundance in response to the spill^10,31^. Subsequent microcosm studies involving the addition of dispersant in seawater showed an increase in the abundance of *Colwellia*, indicating that *Colwellia* is a dispersant degrader^16^. Researchers have been able to isolate *Colwellia* from the water column in the deep sea, and have shown that the organism is capable of oil and dispersant degradation^24,25^. While there have been studies showing the dominance of *Colwellia* in incubations of deep-sea sediments from Faroe-Shetland Channel with oil^26^, much less is known about their role in deep benthic communities than in the water column. The 16S rRNA gene data indicated that the most heavily oiled sediments were enriched in a *Colwellia* species, which was closely related to a *Colwellia* clone from the DWH deep-sea plume^32^. Our study suggests that benthic *Colwellia* plays a role in dispersant degradation.

In this study, ASVs of the genus *Psychrobium* was enriched at both ambient and higher pressures with the addition of oil, and thus, has been defined as an oil responder. The variant that responded to oil at 10 MPa shared 99.07% of sequence identity to an uncultured bacterial clone found in oil-impacted surficial sediment at approximately 1500 meters depth from the Gulf of Mexico^33^. Members of Shewanellaceae have been shown to be piezophilic^34^ and hydrocarbonoclastic^35^. However, the genus *Psychrobium* has not previously been linked to oil degradation. Detailed physiological studies on the preferred hydrocarbons substrates and the biodegradation performance under pressure, of *Psychrobium* could further the understanding on the fate of deposited hydrocarbons in the deep biosphere.

The genus *Moritella* has been identified as a petroleum hydrocarbons degrader in previous studies^26,36,37^ and to increase in relative abundance in response to naphthalene in a seawater microcosm study^36^. In studies using deep-sea sediments, the genus *Moritella* was enriched in the incubations with hydrocarbons mixture containing napththalene and Superdispersant-25^26,37^. In this study, the dispersant-only treatment, using Corexit 9500 A, strongly promoted *Moritella*. This suggested that *Moritella* played a role in the degradation of the dispersant components. Interestingly, several *Moritella* species have been classified as piezophiles, and have optimal growth pressures above 20 MPa^38,39^, but the relative abundance of *Moritella* was consistently lower at 10 MPa than at 0.1 MPa. This reflects the impact of pressure on dispersant biodegradation and may explain a why the degradation of the dispersant slowed down at plume depth after the oil spill^40^.

In addition to *Moritella*, *Thalassotalea* also increased in relative abundance in incubations with dispersant. *Thalassotalea* has been isolated from deep marine environments and is able to grow in the presence oil^41,42^. The major ASV in this study had 99.77% identity to a sequence of a clone found in oil-impacted sediments at the depth of about 1500 m sampled in September 2010 in the Gulf of Mexico^33^. This study is the first to show that *Thalassotalea* may participate in dispersant degradation. Intriguingly, pressure did not exert as strong an effect on *Thalassotalea* as on *Moritella*. The differential effects of pressure on two prominent dispersant responders suggests a different ecophysiology of the two genera. It has been previously reported that the addition of Corexit 9500 increased the number of heterotrophic bacteria in the marine microbial consortium^43^, but the identities of the heterotrophs were not elucidated. Our study demonstrated that *Moritella* and *Thalassotalea* were stimulated by Corexit 9500 A. Thus, *Moritella* and *Thalassotalea* could serve as microbial markers of dispersant application in deep-sea sediment communities.

*Cycloclasticus* is a known obligate aromatic hydrocarbon degrader^44^. The major ASV of *Cycloclasticus* in this study had 97.89% identity to a clone detected in oil-impacted surficial sediment at 1560 m depth of the gulf of Mexico^33^. Consistently higher relative abundance at 10 MPa of *Cycloclasticus* across all treatments suggested that this ASV might be a piezophilic hydrocarbon degrader. Furthermore, this ASV was completely absent in the presence of dispersant at 0.1 MPa (Fig. 5C). Our results suggest that the addition of dispersant may inhibit hydrocarbon degraders growth, as it has been reported for *Marinobacter*^16^. In contrast to the previous study showing that dispersant exacerbated the inhibitory effect of dispersant on hydrocarbon degrading *Rhodococcus*^28^, pressure attenuated the negative impact of dispersant on piezophilic *Cycloclasticus*. This emphasized the pivotal role of hydrostatic pressure as an important factor controlling the fate of oil in the deep sea.

## Conclusion

It is shown that pressure has a significant influence on microbial communities in deep-sea sediments with the addition of oil and dispersant. Hydrostatic pressure differentially impacted microorganisms across different microbial taxa responding to different substrates. Pressure negatively impacted *Moritella*, which responded to dispersant. The slow rate of dispersant biodegradation in the deepwater^40^ and the persistence of the dispersant in the environment^45^ might be explained by the evidence that dispersant degraders are inhibited at high hydrostatic pressure. Therefore, in determining the ultimate fate of the dispersant that is applied in the deep biosphere, the increased pressure and its impact on biodegradation must be considered among the most important environmental factors. Dispersant has been shown to have an inhibitory effect on hydrocarbon degraders^16,28^. The persistence of dispersant due to hydrostatic pressure could further limit oil biodegradation. Therefore, understanding the interplay between high pressure, dispersant, and oil biodegradation, is critical to assess the overall effectiveness and impacts of subsea dispersant application.

## Methods

### Sediment collection and incubation conditions

Sediments were collected during a research cruise on the *RV WeatherBird II* operated by the Florida Institute of Oceanography in August 2016. The 5 sediment coring sites were DWH01 at 1580 m depth, PCB06 at 1043 m depth, DSH08 1123 m depth, DSH10 1490 m depth, and SW01 at 1138 m depth (Supplementary Table S2). The sediment and water samples were stored and shipped to Hamburg University of Technology at 4 °C and atmospheric pressure. Equal wet weights of surficial sediments (0–1 cm) from 5 sites were pooled in a sterile container. Bottom water from 3 sites (DWH01, DSH10, and SW01) were filtered through 0.22 µm filter (Corning, USA) and added to the pooled sediments to make a sediment slurry of 50 mg of sediment per ml. Incubations containing only 5 ml of the slurry served as controls. Experimental treatments included the addition of the following added in sterile 10 ml glass vials: (1) 50 µl of autoclaved light Louisiana sweet crude oil, (2) 2 µl dispersant (Corexit 9500 A, Nalco Chemical company), or (3) oil and dispersant (1:25 volumetric ratio of dispersant to oil). Treatments and controls were run in 6 replicates. The incubations were conducted at atmospheric pressure (0.1 MPa) and elevated pressure (10 MPa). The 10 MPa pressure treatment is comparable to the pressure where the subsea plume deposited hydrocarbons during the Deepwater Horizon spill^6^. Pressure of 10 MPa was achieved by continuously pressurizing with nitrogen gas into the headspace containing atmospheric air in order to keep the aerobic condition as previously described^21,22^. The slurries were incubated at 4 °C with stirring at 200 rpm by means of a magnetic stirring bar for 7 days.

### DNA extraction and 16S rRNA amplicon sequencing

Total DNA was extracted from 2 ml of sediment slurry with PowerSoil DNA Isolation Kit (QIAGEN, Germany) according to manufacturer’s protocol. Paired ended amplicon sequencing of V3 and V4 variable regions of the 16S rRNA gene was performed at LGC Genomics (Germany) facility on the Illumina MiSeq platform using 341 F (CCTACGGGNGGCWGCAG)^46^ and 785 R (GACTACHVGGGTATCTAAKCC)^47^ primers. The resulting sequences were truncated, quality-filtered, denoised, chimera-filtered and merged with DADA2^48^ as implemented in QIIME2 (2018.4 Release)^49^. The taxonomy was assigned to ASVs with the naive-Bayes classifier^50^. The classifier was trained with SILVA132 released database^51^ trimmed to V3-V4 region^52,53^.

### Statistical analysis

The resulting sequences were exported into and further processed in the Phyloseq package^54^ in R^55^. The samples were rarefied to even sequence depth across samples to a minimum number where the rarefaction curves were constant (8181 sequences per sample). The sequence abundances were transformed into relative abundances by dividing by total reads. The relative abundances were plotted with ggplot2^56^. The statistical analyses of alpha diversity and beta diversity were calculated using Phyloseq. The differences between the communities was determined using the function adonis in the package Vegan^57^. The permutational multivariate analysis of variance (PERMANOVA) using Bray-Curtis matrix were carried out with 999 permutations. Differential abundant taxa between pressures were analyzed using Calour with nominal discrete false discovery rate of q < 0.1^58^. Each treatment (oil/dispersant/both oil and dispersant) was first compared to the controls at the same pressure to identify ASVs that responded to the treatment. The 6 resulting groups of ASVs were subjected to Venn diagram analysis using InteractiVenn^59^ to identify the ASVs that responded to oil, dispersant, or dispersed oil at 0.1 MPa or at 10 MPa.

## Supplementary information

Supplementary information.

## Data Availability

Sequences from this study are available through the European Nucleotide Archive under project PRJEB33386 (ERP116173).
