# Supplementary material for: Influence of oil, dispersant, and pressure on microbial communities from the Gulf of Mexico
Source: Sci Rep. 2020 Apr 27;10:7079. doi: 10.1038/s41598-020-63190-6 (PMC7184722; doi:10.1038/s41598-020-63190-6)
Supplement: Supplementary file 1 — Supplementary information. [file 41598_2020_63190_MOESM1_ESM.pdf]

Supplementary Information

The influence of oil, dispersant, and pressure on microbial communities from the Gulf of Mexico

Nuttapol Noirungsee[1], Steffen Hackbusch[1], Juan Viamonte[1], Paul Bubenheim[1], Andreas Liese[1], and Rudolf Müller[1,*]

[1]Hamburg University of Technology, Institute of Technical Biocatalysis, Hamburg, 21073, Germany

*Corresponding author ru.mueller@tu-harburg.de

Contents

Supplementary Figure S1. Venn diagram of shared differentially abundant ASVs between treatment and pressure.

Supplementary Figure S2. Venn diagram of shared differentially abundant ASVs between treatment and pressure.

Supplementary Table S1. The results of differentially abundant analysis and Venn diagram analysis showing the number of variants which were specifically enriched in the sample at a given condition

Supplementary Table S2 Locations and depths of sediment collection site

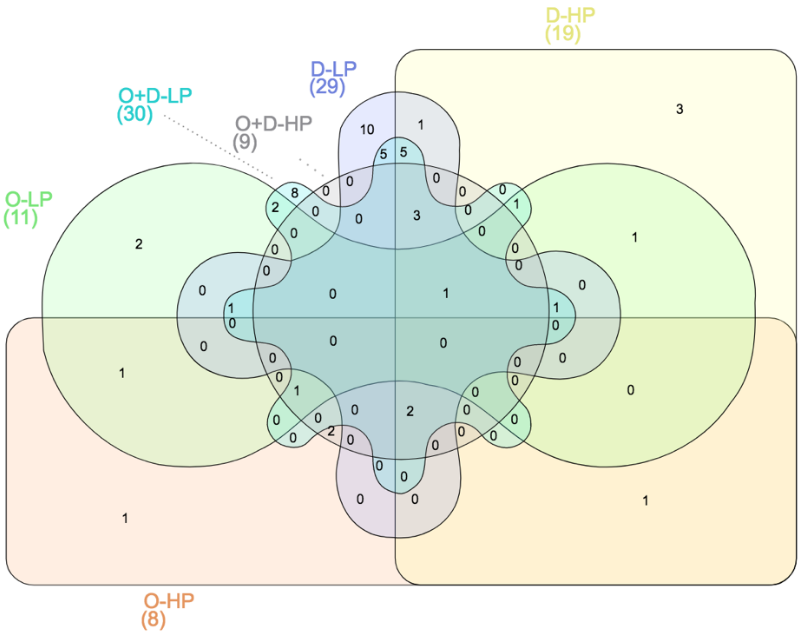

Supplementary Figure S1. Venn diagram of shared differentially abundant ASVs between treatment and pressure. Each treatment (O: Oil only; D: Dispersant only; O+D: Oil and Dispersant) was first compared to the control at the respective pressure (LP: 0.1 MPa; HP: 10 MPa) to detect ASVs that were enriched by treatment. The enriched ASVs were then included in the Venn diagram analysis.

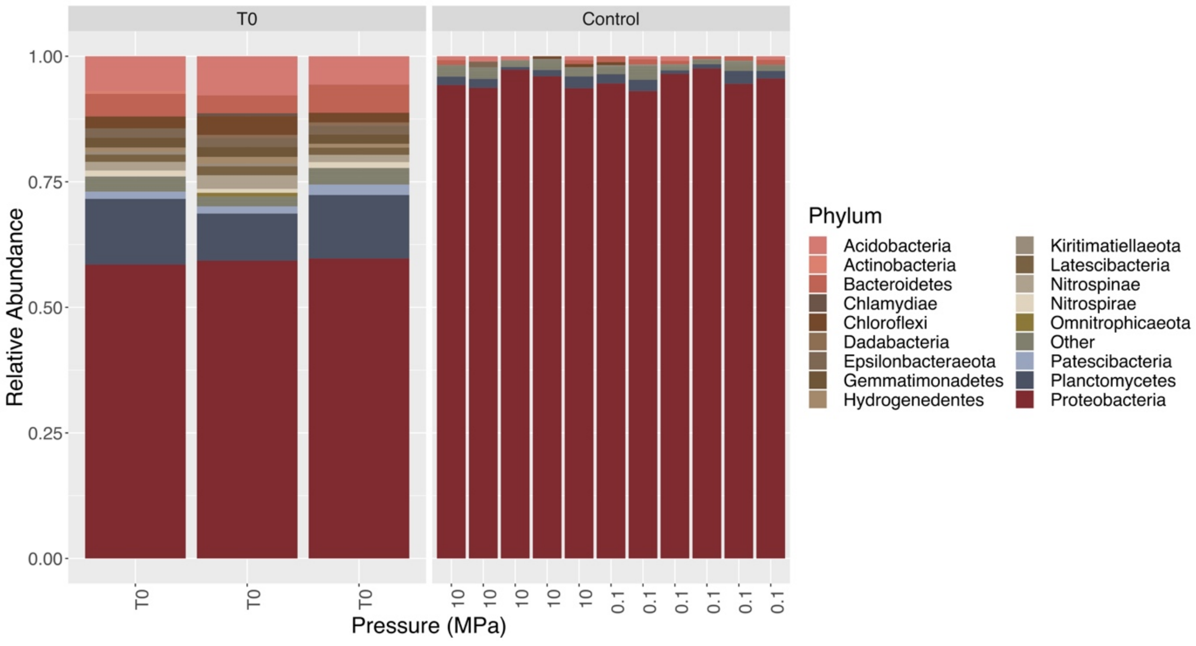

Supplementary Figure S2. Relative abundance plots showing the initial communities before incubations and Control (no dispersant or oil addition). Taxa shown were grouped at phylum level. Each single bar represents one biological replicate.

Supplementary Table S1. The results of differentially abundant analysis and Venn diagram analysis showing the number of variants which were specifically enriched in the sample at a given condition

**Family**	**Genus**	**Number of Variants Enriched**					
		**Pressure (MPa)**					
		**Oil**		**Dispersant**		**Dispersed oil**	
		**0.1**	**10**	**0.1**	**10**	**0.1**	**10**
Shewanellaceae	*Psychrobium*	2	3	3	0	0	0
Colwelliaceae	*Colwellia*	0	0	6	0	0	0
Moritellaceae	*Moritella*	0	0	1	0	0	0
Alteromonadaceae	*Alkalimarinus*	0	0	1	0	0	0
Halomonadaceae	*Cobetia*	0	0	1	0	1	0
Nitrincolaceae	*Motiliproteus*	0	0	1	0	2	0
Colwelliaceae	*Thalassotalea*	0	0	0	2	0	0
Endozoicomonadaceae	*Endozoicomonas*	0	0	0	1	0	0
Rhodobacteraceae	*Roseobacter*	0	0	0	1	0	0
Nitrincolaceae	*Amphritea*	0	0	0	0	3	0
Other	*Other*	2	0	0	1	1	0

Supplementary Table S2 Locations and depths of sediment collection site

Site	Latitude (N)	Longitude (W)	Water depth (m)
DWH01	28 43.457	88 23.205	1580
PCB06	29 5.99	87 15.93	1043
DSH08	29 7.37	87 52.088	1123
DSH10	28 58.764	87 53.480	1490
SW01	28 14.462	89 7.162	1138
